# Motivation to Learn and Multilingualism across the Adult Life Stages in the U.S.

**DOI:** 10.1093/geroni/igab046.3414

**Published:** 2021-12-17

**Authors:** Shalini Sahoo, Takashi Yamashita, Roberto Millar, Phyllis Cummins

**Affiliations:** 1 University of Maryland, Baltimore, Hanover, Maryland, United States; 2 University of Maryland, Baltimore County, Baltimore, Maryland, United States; 3 The Hilltop Institute, Baltimore County, Maryland, United States; 4 Miami University, Oxford, Ohio, United States

## Abstract

Lifelong learning or continuing education over the life course has become necessary to navigate a rapidly changing technological landscape. Motivation to learn (MtL) is essential for facilitating lifelong learning. In the U.S., most of the educational opportunities are available in English. Moreover, little is known about associations between being multilingual and MtL across the life stages. This study analyzed nationally representative data from the 2012/2014/2017 Program for International Assessment of Adult Competencies (PIAAC) restricted use file (RUF). Using a previously established latent MtL construct, structural equation models were estimated by four age groups --- 25-34 (n = 2,310); 35-44 (n = 1,610); 45-54 (n = 1,670); and 55 and older (n = 2,620). Results showed that being multilingual was associated with greater MtL among younger age groups, including age 25-34 (b = 0.20, p = 0.01) and 35-44 (b = 0.28, p < 0.001), after adjusting for the demographic, socioeconomic and health characteristics of individuals. Multilingualism was not associated with MtL among older age groups, including 45-54 (b = 0.06, p = 0.50) and 55 and older (b = 0.13, p = 0.19). Findings suggest that education policies that target younger multilingual adults are likely to be effective while enhancing MtL of monolingual (i.e., English-speaking only) adults seems to be a necessary first step. Yet, a similar approach may not be effective for older adults, arguably due to more diverse life circumstances, educational needs, and learning style preferences. More detailed interpretations of empirical results and theoretical explanations are needed.

